# Self-reported campus alcohol policy and college alcohol consumption: a multilevel analysis of 4592 Korean students from 82 colleges

**DOI:** 10.1186/s13011-020-0255-9

**Published:** 2020-02-01

**Authors:** Sarah Soyeon Oh, Yeong Jun Ju, Sung-in Jang, Eun-Cheol Park

**Affiliations:** 1 Institute of Health Services Research, Yonsei University, Seoul, Republic of Korea; 2 Department of Public Health, Graduate School, Yonsei University, Seoul, Republic of Korea; 3 Department of Preventive Medicine, Ajou University College of Medicine, Gyeonggi Suwon, Republic of Korea; 4 Department of Preventive Medicine, Yonsei University College of Medicine, 50 Yonsei-ro, Seodaemun-gu, Seoul, 120-752 South Korea

**Keywords:** College drinking, College alcohol policy, Audit-c, Campus policy, Alcohol education

## Abstract

**Background:**

Campus alcohol policy has been associated with student alcohol consumption in numerous studies. However, more information is required to assess the extent to which school policy affects student drinking behavior; especially when both individual-level sociodemographic characteristics of students and area-level characteristics of college campuses are controlled for. Thus, this paper explores the association between campus alcohol policy and student alcohol consumption among a nationally representative sample of college students in South Korea, while controlling for both individual and area-level characteristics.

**Methods:**

We surveyed and analyzed the data of 4592 students from 82 colleges. Multilevel (hierarchical) linear modeling was used to identify the association between campus alcohol policy and alcohol consumption levels, measured via the AUDIT-C (Alcohol Use Disorders Identification Test – Consumption). Controlled individual-level characteristics included sex, year level, major, GPA (grade point average), pocket money, smoking status, stress level, depressive thoughts, suicidal thoughts, and number of clubs/organizations. Controlled area-level characteristics included college type, number of students, number of faculty members, number of workers/administrators, and region.

**Results:**

Compared to students unaware of their school’s campus alcohol policy, students who self-reported that their campuses allow drinking in outdoor spaces (β = 0.755 *p* = 0.010) or in all areas (β = 0.820, *p* = 0.044) had higher AUDIT-C scores. Students attending schools with a large number of students, males, freshmen, students with low GPA, students with high amounts of pocket money, and smokers also had higher alcohol consumption scores relative to their peers. Alcohol education experience in the form of lectures, mail, and/or campaigns were not associated with student alcohol consumption levels.

**Conclusion:**

Our results suggest an association between self-reported campus alcohol policy and student alcohol consumption. College educators and administrators must be aware that relative to students unaware of their school’s campus alcohol policy, students at colleges that allow drinking in outdoor spaces or all areas consume higher amounts of alcohol than their peers; even when area-level factors are controlled for.

**Trial registration:**

Yonsei IRB (IRB number: Y-2017-0084). https://irb.yonsei.ac.kr Date of registration: 01/2017. Date of enrolment of first participant to trial: 03/01/2017. Y-2017-0084.

## Background

Alcohol use among college students is problematic globally, but little is known about the extent to which various measures, such as campus alcohol policy and education, deter excessive student drinking. In the context of South Korea, transition to college is often associated with an escalation in binge drinking; one investigation reported that approximately 71.2% of students consume at least four to five standard drinks per drinking session [1]. In the United States, although binge drinking rates have decreased over time, 30–40% of adults consume four to five standard drinks per drinking session [2], while in Europe, around 60% of men and 41% of women between the ages of 18 to 23 binge drink regularly [3]. Considering that there are numerous negative consequences of college binge drinking including violence, date rape, accidents, and academic problems [4], more research on prevention efforts is necessary.

Rates of student alcohol use have been shown to vary between schools; even when individual-level characteristics such as gender, race, and ethnicity [5] have been adjusted for. There are also multiple area-level characteristics such as peer drinking norms [6], wealth and entertainment of the neighborhood surrounding the campus [7], and alcohol outlet density [8, 9] that previous studies have associated with student drinking.

The National Institute on Alcohol Abuse and Alcoholism (NIAAA) has identified the following environmental-focused strategies for decreasing college binge drinking: 1) retaining the minimum legal drinking age (MLDA) of 21, 2) enforcing the MLDA, 3) increasing taxes on alcohol, 4) retaining a ban on Sunday alcohol sales, and 5) enacting bans on happy hours and other price promotions. Although such policies are enacted at the state or local level, when colleges partner with other organizations or coalitions to implement or retain such policies, reductions in risky alcohol use and related problems among students are possible [10].

Previous studies in the international literature have also shown that certain policy interventions influence student alcohol consumption. Whilst policies vary among institutions, emerging evidence indicates that policy-makers should target both individual and environmental strategies to reduce excessive alcohol consumption and binge drinking among students. Typical environmental policies include campus alcohol bans, bans for minors, no alcohol use at college events, prohibition of beer kegs and alcohol displays on campus [11], and limitations on maximum number of drinks purchasable per student [12].

In one study, students attending schools with a ban on alcohol use were up to 30% less likely to engage in binge drinking [13]. Attending colleges that restrict high volume sales or target underage drinking has been associated with lower rates of alcohol-involved driving while substance-free residence halls have been associated with reduced alcohol-related problems [14]. Regulating excessive alcohol use, through distance and access-based interventions that reduce the average distance between a college and outlet or the number of outlets in a county have also been effective in decreasing excessive alcohol consumption [15].

Banning alcohol advertisements and kegs on campus, and enforcing deterrence policies more strictly have been associated with decreased alcohol consumption [16]. Recent studies have also found that while campaigns to drink responsibly are ineffective for heavy drinkers, strategic campaigns that promote responsible drinking may be effective among mild and moderate drinkers [17]. However, in 2008, Nelson and colleagues found that 23% of colleges in the United States were not employing any recommended strategies to reduce alcohol-related harm, while 45% were only employing a single recommended strategy such as 1) interventions challenging alcohol expectancies, 2) restrictions on alcohol retail outlet density, 3) enforcement of laws to prevent alcohol-impaired driving, and/or 4) responsible beverage service policies in social and commercial settings [18]. Overall, limited research has been done to assess the association between self-reported campus alcohol policy and education experience on alcohol consumption among college students.

On the individual level, alcohol education programs, especially those directed towards individuals that typically use alcohol at higher rates (e.g. members of Greek organizations and participants of athletic events) have shown to be effective in some studies [19]. Ultimately, according to a study of 734 college administrators, most institutions in the United States continue to offer some type of alcohol education program, despite their limited success, in combination with restrictive environmental policies that reduce student access to alcohol (e.g. limits on alcohol deliveries, and/or alcohol advertisements on campus) [11].

Although alcohol policies have been enacted to prevent and reduce harmful drinking of college students, limited research has been done to assess the association between type of campus alcohol policy and student alcohol consumption from a multilevel model approach. Therefore, the present study focuses on examining the association between perceived college alcohol policy and student drinking, while controlling for both individual-level and college-level characteristics.

## Methods

### Study sample and data

In the 2017 national statistics published by the Korean Educational Development Institute on college students, we found that 1,951,940 students (4-year: 1,506,745; liberal arts: 445,195) are enrolled in 356 colleges (4-year: 195, liberal arts: 161) in South Korea. Thus, we stratified a proportionately representative sample of undergraduate students from 54 4-year colleges and 28 liberal arts colleges (Table 1). Students in these colleges were further stratified according to sex, year level, major, GPA, pocket money, smoking status, stress level, depressive thoughts, suicidal thoughts, and number of clubs/organizations.

**Table 1:** Stratification of a nationally representative sample of college students in South Korea

	4-year Colleges		2-year Colleges		Total
	Population ratio (%)	Students(colleges)	Population ratio (%)	Students(colleges)	Students(colleges)
Seoul	12.6	630 (10)	4.2	210 (3)	840 (13)
Incheon/Gyeonggi	9.4	471 (8)	8.1	403 (7)	874 (15)
Gangwon	5.6	280 (5)	2.5	124 (2)	404 (7)
Daejeon/Chungjeong	11	552 (9)	4.7	233 (4)	785 (13)
Gwangju/Jeolla	8.5	426 (7)	4.9	245 (4)	671 (11)
Daegu/Gyeongbuk	8.3	417 (7)	5.4	270 (4)	687 (11)
Busan/Ulsan/Gyeongnam	9.8	488 (8)	5	251 (4)	739 (12)
Total	65.3	3264 (54)	34.7	1736 (28)	5000 (82)

In total, 5000 students completed the survey instrument. The response rate was 68.7%, with the total number of approached participants being 7278. A financial incentive of 10,000 Korean Won (equivalent to around 9 U.S. Dollars) was given to each participant upon completion of the 14-page survey instrument. Of these students, we excluded 10 students who had missing data for their major and GPA, and 211 students who had missing data for questions related to the AUDIT-C, for a final study sample of 4592 college students. More information regarding the survey have been published in previous studies [20, 21].

Data was collected via face-to-face surveys with students. Questions were mainly about student drinking behavior, health, sociodemographic characteristics, and thoughts on campus-alcohol policy. Whenever possible, the instrument included alcohol-related questions that had been previously used in other international, national or large-scale epidemiological studies including the Harvard College Alcohol Study [14], the Korea National Health and Nutrition Examination Survey (KNHANES) [22], and the Korea Youth Risk Behavior Web-Based Survey (KYRBS) [23]. College-level information such as number of students, faculty, and staff were found on the Korean Educational Development Institute website, which provides basic information about all registered colleges in the country.

A standard drink was defined as the amount of alcohol contained in one glass of alcohol drink (approximately 8 g of pure alcohol), equivalent to:1 shot of soju, 1 glass of bottled beer, 2/3 of a canned beer, 1/2 glass of draft beer, 1/2 bowl of makgeolli (rice wine), 1/2 glass of wine, 1 glass of whiskey, 1 shot of cheongju (refined rice wine), 1 shot of herbal liquor, 1 shot of fruit wine, or a 3/5 glass of mixed liquor (soju+beer), in accordance with the standards of the Korea Centers for Disease Control & Prevention.

Our survey instrument followed the guidelines of the Institutional Review Board of Yonsei University’s College of Medicine (Number: Y-2017-0084). All procedures were performed in accordance with the ethical standards of the Declaration of Helsinki. Informed consent was obtained from all individual participants included in the survey in written form. Data collectors were trained about the survey’s ethical standards regarding privacy, anonymity, and confidentiality by our research team and collaborators from Gallup Korea. Each question of the questionnaire was required to be administered privately to students in a face-to-face manner at a quiet, enclosed space on campus such as a café or lecture room. The survey contained no identifying values that could link the information to the participant; making it completely impossible for researchers to identify specific participants.

### Measures

#### Outcome variable

Alcohol intake, measured through the Alcohol Use Disorders Identification Test-Consumption (AUDIT-C), was selected as the outcome variable. The AUDIT-C is an abbreviated 3-item measure consisting of the first three questions from the full-length AUDIT questionnaire (Table 2). The AUDIT-C assesses alcohol consumption over the past year, and can help identify persons who are hazardous drinkers or have active alcohol use disorders (including alcohol abuse or dependence). Items are scaled (scale: 0–4) and summed to create a total score (scale: 0–12). Higher AUDIT-C scores indicate greater alcohol consumption; generally, the higher the AUDIT score, the more likely the patient’s drinking is affecting his or her safety. Among Korean men, a score of 4 or more is considered positive, optimal for identifying hazardous drinking or active alcohol use disorders, whilst a score of 3 or more is considered positive among Korean women [24].

**Table 2:** AUDIT-C Questionnaire

	Scoring System^a^				
	0	1	2	3	4
1. How often do you have a drink containing alcohol?	Never	Monthly or less	2–4 times per month	2–3 times per week	4+ times per week
2. How many standard drinks containing alcohol do you have on a typical day?	1–2	3–4	5–6	7–9	10+
3. How often do you have 6 or more drinks on one occasion?	Never	Less than monthly	Monthly	Weekly	Daily or almost daily

^a^Scoring: Sum of 3 questions result in possible AUDIT-C score of 0–12 points with recommended screening thresholds: ≥4 for men; ≥3 for women

### Perceived type of campus alcohol policy

Perceived type of campus alcohol policy was measured via individual answers to the question, “What is your university’s campus alcohol policy?” Response options were as follows: “Unaware of campus alcohol policy,” “bans all drinking on campus,” “bans minors (under 19) from drinking on campus,” “occasionally allows drinking on campus at certain locations/during events,” “allows drinking in outdoor spaces,” and “allows drinking in all areas.” Students could only select one answer, based on their knowledge of their school’s campus alcohol policy. Multiple choice options were unavailable.

### Alcohol education experience (lecture/mail/campaign)

Alcohol education experience was measured via individual answers to the question, “In the past 12 months, how many times have you encountered or taken part in the following alcohol prevention activities at you university?” Response options were as follows: “attended alcohol prevention programs, lectures, or training,” “received a mail or brochure on alcohol prevention,” “saw posters or promotion materials on alcohol prevention,” and “participated in a moderation campaign on campus.” Individuals were able to select their response on the following frequency scale: “never,” “once,” “twice,” “3 times,” “4 or more times.” The sum of these responses was re-categorized into a new variable: ‘alcohol education experience’ and individuals were classified into the following categories: “none,” “1–2 times,” “3–4 times,” “more than 5 times.”

### Statistical analysis

In order to examine the study participants’ general characteristics, chi-square tests were performed to compare differences between groups. To examine the association between perceived type of campus alcohol policy and alcohol education experience with alcohol consumption, multilevel linear regression analysis was employed. Both area-level characteristics with respect to each college campus (college type, number of students, number of faculty members, number of workers/administrators, region) and individual-level characteristics (sex, year level, major, GPA, pocket money, smoking status, stress level, depressive thoughts, suicidal thoughts, number of clubs/organizations) were controlled for in the mixed model.

The beta values used in this model indicate the non-standardized regression coefficient, which signifies how much the mean of the dependent variable (AUDIT-C) changes given a one-unit shift in the independent variable (perceived type of campus alcohol policy / frequency of alcohol education experience). Kendall’s tau-b correlation coefficient was used to measure the relationship between our variables of interest (perceived policy type, alcohol education experience frequency) and AUDIT-C.

The simultaneous relationship between type of campus alcohol policy and alcohol education experience frequency on AUDIT-C was determined through subgroup analyses, by running the linear regression analysis on the sample, when stratified by sex. The calculated *p*-values in this study were considered significant if lower than 0.05. All analyses were performed using SAS software, version 9.4 (SAS Institute, Cary, North Carolina, USA).

## Results

Table 3 shows the general characteristics of the study sample. Three thousand five hundred ninety-six students reported to being “unaware of campus alcohol policy,” followed by 704 students reporting to college “bans all drinking on campus,” 85 students reporting to college “bans minors from drinking on campus, 201 students reporting to college “occasionally allows drinking on campus at certain locations/during events, 148 students reporting to college “allows drinking in outdoor spaces,” and 69 students reporting to college “allows drinking in all areas.” Students who perceived that their campuses allow drinking in outdoor spaces (7.189 ± 3.009) or all areas (7.232 ± 3.392) had the highest AUDIT-C scores.

**Table 3:** General characteristics of campus alcohol policy and alcohol consumption

	AUDIT-C Score			
	N	Mean	SD	*p*-value
Area-level Characteristics (*n* = 82)				
College type				
College (four-year)	47	6.403	3.329	0.083
Technical (three-year)	15	6.476	3.300	
Technical (two-year)	10	6.231	3.293	
Other^a^	10	6.029	3.212	
Number of Students				
Q1 (Low)	21	6.314	3.243	0.000
Q2	20	6.067	3.346	
Q3	21	6.447	3.302	
Q4 (High)	20	6.644	3.328	
Number of Faculty Members				
Q1 (Low)	21	6.449	3.236	0.006
Q2	20	6.080	3.315	
Q3	21	6.442	3.395	
Q4 (High)	20	6.503	3.277	
Number of Workers/Administrators				
Q1 (Low)	21	6.491	3.229	0.090
Q2	20	6.167	3.347	
Q3	21	6.429	3.427	
Q4 (High)	20	6.368	3.231	
Region				
Metropolis^b^	37	6.278	3.358	0.092
Town/Country^c^	45	6.440	3.267	
Individual-level Characteristics (*n* = 4592)				
Perceived Type of Campus Alcohol Policy				
Unaware of campus alcohol policy	3596	6.319	3.323	0.011
Bans all drinking on campus	704	6.314	3.373	
Bans minors (under 19) from drinking on campus	85	6.424	2.718	
Occasionally allows drinking at certain locations/during events	201	6.448	3.181	
Allows drinking in outdoor spaces	148	7.189	3.009	
Allows drinking in all areas	69	7.232	3.392	
Alcohol Education Experience (Lecture/Mail/Campaign)				
None	3797	6.359	3.342	0.705
1–2 times	705	6.465	3.182	
3–4 times	122	6.238	3.387	
More than 5 times	179	6.179	3.065	
Sex				
Male	2356	6.764	3.222	<.0001
Female	2447	5.982	3.349	
Year Level				
1	1502	6.459	3.284	0.001
2	1540	6.553	3.281	
3	840	6.186	3.349	
≥ 4	921	6.062	3.339	
Major				
Humanities and Social Sciences	2279	6.262	3.342	0.054
Engineering/Natural Sciences	1956	6.504	3.244	
Liberal Arts	568	6.305	3.394	
GPA				
≥ 4.0	705	6.172	3.390	<.0001
3.5–4.0	1709	6.098	3.365	
3.0–3.5	1698	6.444	3.234	
≤ 3.0	691	7.030	3.178	
Pocket Money				
Q1 (Low)	1739	5.704	3.323	<.0001
Q2	1259	6.274	3.260	
Q3	940	6.843	3.286	
Q4 (High)	865	7.309	3.070	
Smoking Status				
Current Smoker	1092	7.649	2.941	<.0001
Past Smoker	217	7.788	2.686	
Non-Smoker	3494	5.876	3.322	
Stress Level				
High	540	6.106	3.373	0.085
Normal	3474	6.424	3.301	
Low	789	6.283	3.299	
Depressive Thoughts				
Yes	555	6.501	3.193	0.304
No	4248	6.347	3.325	
Suicidal Thoughts				
Yes	138	6.370	3.283	0.987
No	4665	6.365	3.311	
Number of clubs/organizations				
None	2458	6.317	3.310	0.126
One	1879	6.356	3.323	
Two or more	466	6.657	3.247	
Total	4803	6.365	3.310	

^a^Cyber college, vocational school, technical school

^b^Seoul, Busan, Daegu, Incheon, Gwangju, Daejeon

^c^Gyeonggi, Gangwon, Chungbuk, Chungnam, Jonbuk, Jonnam, Gyeongbuk, Gyeongnam

Kendall’s correlations showed that AUDIT-C was positively correlated with both perceived policy type (Kendall’s tau-b = 0.24, *p* < .0001) and education experience (Kendall’s tau-b = 0.02, *p* = 0.04). Kendall’s correlations also showed that AUDIT-C was positively correlated with both perceived policy type and education experience. Considering that small correlation coefficients can be highly significant in large sample sizes and a Kendall correlation is equal to 2/π times the inverse sine of the Pearson correlation [25], these correlation coefficient values may represent significant associations.

Table 4 shows the results of the multilevel regression analysis performed to investigate the association between various factors and AUDIT-C score among our study sample. Compared to students “unaware of campus alcohol policy,” students whose campuses “allow drinking in outdoor spaces” (β = 0.755, *p =* 0.290) or “allow drinking in all areas” (β = 0.820, *p =* 0.044) on campus had higher AUDIT-C scores, even when area-level characteristics including college type, number of students, number of faculty members, number of workers/administrators, and college region were controlled for.

**Table 4:** Results of the multilevel regression analysis analyzing campus policy and alcohol consumption

	AUDIT-C Score		
	β	S.E	*p*-value
Intercept	5.020	0.307	<.0001
Area-level Characteristics (*n* = 82)			
College type			
College (four-year)	Ref.		
Technical (three-year)	−0.127	0.400	0.876
Technical (two-year)	−0.294	0.440	0.137
Other^a^	−0.466	0.400	0.088
Number of Students			
Q1 (Low)	Ref.		
Q2	−0.009	0.347	0.516
Q3	0.506	0.439	0.011
Q4 (High)	0.955	0.552	0.003
Number of Faculty Members			
Q1 (Low)	Ref.		
Q2	−0.185	0.502	0.118
Q3	−0.007	0.703	0.761
Q4 (High)	−0.031	0.874	0.593
Number of Workers/Administrators			
Q1 (Low)	Ref.		
Q2	−0.489	0.471	0.036
Q3	−0.281	0.608	0.385
Q4 (High)	−0.920	0.669	0.008
Region			
Metropolis^b^	Ref.		
Town/Country^c^	0.331	0.216	0.048
Individual-level Characteristics (n = 4592)			
Perceived Type of Campus Alcohol Policy			
Unaware of campus alcohol policy	Ref.		
Bans all drinking on campus	0.123	0.174	0.481
Bans minors (under 19) from drinking on campus	0.237	0.361	0.512
Occasionally allows drinking at certain locations/during events	0.150	0.260	0.565
Allows drinking in outdoor spaces	0.755	0.290	0.010
Allows drinking in all areas	0.820	0.405	0.044
Alcohol Education Experience (Lecture/Mail/Campaign)			
None	Ref.		
1–2 times	0.003	0.147	0.986
3–4 times	−0.113	0.300	0.708
More than 5 times	−0.286	0.255	0.265
Sex			
Male	Ref.		
Female	−0.269	0.114	0.021
Year Level			
1	Ref.		
2	−0.068	0.130	0.603
3	−0.512	0.156	0.001
≥ 4	−0.614	0.158	0.000
Major			
Humanities and Social Sciences	Ref.		
Engineering/Natural Sciences	0.068	0.125	0.587
Liberal Arts	−0.097	0.173	0.573
GPA			
≥ 4.0	Ref.		
3.5–4.0	−0.032	0.149	0.831
3.0–3.5	0.242	0.151	0.109
≤ 3.0	0.468	0.178	0.009
Pocket Money			
Q1 (Low)	Ref.		
Q2	0.677	0.126	<.0001
Q3	1.174	0.140	<.0001
Q4 (High)	1.534	0.144	<.0001
Smoking Status			
Current Smoker	1.456	0.131	<.0001
Past Smoker	1.543	0.226	<.0001
Non-Smoker	Ref.		
Stress Level			
High	Ref.		
Normal	0.296	0.155	0.058
Low	0.176	0.189	0.353
Depressive Thoughts			
Yes	0.197	0.166	0.239
No	Ref.		
Suicidal Thoughts			
Yes	−0.216	0.296	0.470
No	Ref.		
Number of clubs/organizations			
None	Ref.		
One	0.073	0.112	0.515
Two or more	0.337	0.175	0.055

^a^Cyber college, vocational school, technical school

^b^Seoul, Busan, Daegu, Incheon, Gwangju, Daejeon

^c^Gyeonggi, Gangwon, Chungbuk, Chungnam, Jonbuk, Jonnam, Gyeongbuk, Gyeongnam

Alcohol education experience was not a predictor of reduced alcohol consumption. Females (β = − 0.269 *p* = 0.021) scored lower on the AUDIT-C than males as did seniors (β = − 0.614, *p =* 0.003 = 0) relative to freshmen. Students in the lowest GPA bracket (≤3.0) scored higher on the AUDIT-C than students in the highest GPA bracket (≥4.0). Past smokers (β = 1.543, *p* < .0001) and students who reported to currently smoking (β = 1.456 p < .0001) had higher AUDIT-C scores compared to those reporting to not smoking, as did students participating in two or more clubs/organizations (β = 0.337, *p* = 0.055) relative to no clubs/organizations.

**Fig. 1:**
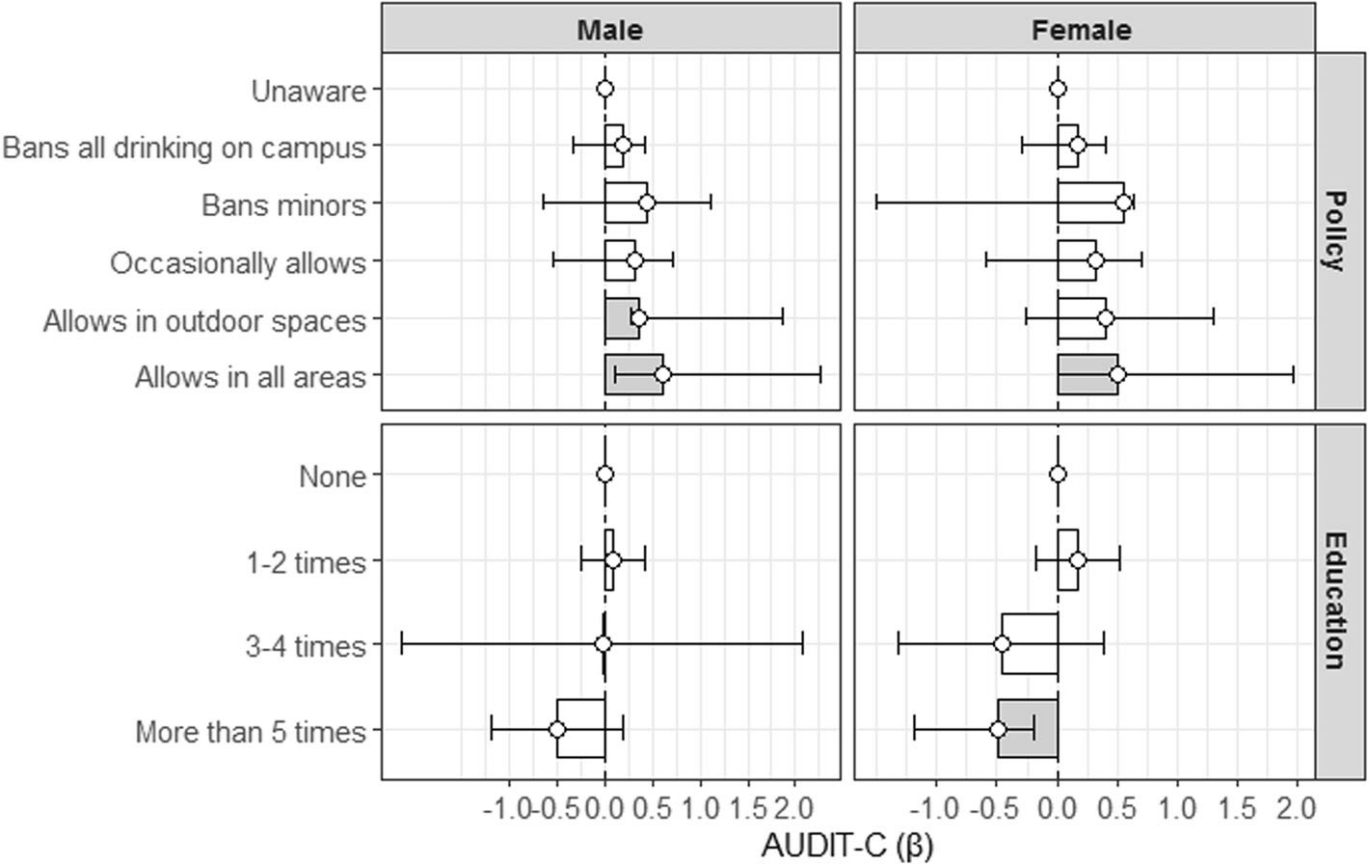
Subgroup analysis of the association between perceived alcohol policy and education experience by sex

For males, allowing alcohol consumption in outdoor spaces (β = 1.1690, *p =* 0.0009) or in all areas (β = 1.0777, *p* = 0.0479) resulted in more alcohol consumption (Fig. 1). For females, allowing alcohol consumption in all areas (β = 0.9834, *p* = 0.0486) resulted in more alcohol consumption. Receiving alcohol education 1–2 times, or 3–4 times were not associated with higher AUDIT-C, which was in alignment with the existing body of literature.

## Discussion

Our results suggest an association between self-reported campus alcohol policy and student alcohol consumption. Relative to students unaware of campus alcohol policy, students who believe that their college allows drinking in outdoor spaces or all areas may consume higher amounts of alcohol than their peers. Such findings are in alignment with previous studies that have found that students drink more on school grounds when they perceive lax policy enforcement by college officials [26].

Interestingly, alcohol education experience, pertaining to alcohol prevention programs through lectures, mail, brochures, posters, promotion materials, or moderation campaigns, was not a significant predictor of decreased alcohol consumption among students in our investigation, and only affected female students who had received alcohol education more than five times in their college years. Alcohol education programs have had mixed results when it comes to college interventions: online and/or offline alcohol education courses for college students have been both successful [27] and unsuccessful [16, 28, 29] in mitigating alcohol-related high-risk behaviors among student populations. What is clear is that while educational experiences may have no effect among all college students, among students who violate campus alcohol policies and/or engage in high-risk drinking behaviors, alcohol education or counseling is an effective measure in preventing alcohol misuse [30]. Furthermore, as emphasized by Kelly-Weeder and colleagues, integrating educational interventions with environmental approaches can increase program effectiveness [17].

Our findings also show specific socio-demographic groups that should be particularly targeted when establishing campus alcohol policies: males, freshmen, students with low GPA, students receiving high amounts of pocket money, current and past smokers, and students in two or more clubs/organizations. Findings from major college alcohol investigations including the Harvard College Alcohol Study have already noted these vulnerable populations; as seen in the 1993, 1997, 1999, and 2001 Harvard College Alcohol Study, males, students under the age of 21, students with academic problems related to alcohol such as missing class and/or getting behind in school work [13], and smokers have all been associated with consuming more amounts of alcohol than other subpopulations [4]. Likewise, previous studies have found that students who are more active in school activities such as clubs/organizations [31] or university athletics drink more, and find alcohol problematic on campus [15].

A difference between previous studies and our study is that in the context of year level, students in South Korea have the highest AUDIT-C scores in their freshman year, whereas students in international studies mostly consume high amounts of alcohol during their sophomore and junior years [3, 13, 32, 33]. This phenomenon may be particular to South Korea; students in South Korea have been noted to consume the most amount of alcohol in their freshman year (often, unwillingly) at various orientation, and/or freshmen events where juniors and seniors pressure incoming students to drink [1]. Similar trends were also found among some Asian countries like China [32] and Taiwan [34], where alcohol use was greatest among 1st year students. However, in most European countries like France [33], Belgium, Colombia, Ireland, and Poland [3, 4] age and/or year of study were not associated with binge drinking and associated drinking behaviors.

Our study has a number of limitations. First, our study is cross-sectional in design and therefore, it is difficult to make causal inferences about the effect of campus alcohol policy type or education experience on alcohol consumption. The data is based on self-reported answers, and the question about campus alcohol policy may be ambiguous as only single choice answers were possible. Furthermore, the group size is too small to meaningfully interpret trends, especially because being ‘unaware’ of campus alcohol policy does not mean full prohibition or liberalization. Future investigations should attempt to overcome these limitations through the survey instrument and design.

Second, there are not enough previous studies with regard to a nationally representative sample of Koreans when it comes to measuring type of campus alcohol policy/education experience and its effect on drinking behavior of college students. It is difficult to see whether the values we calculated are similar to that of the statistics found in previous studies, especially for the college students’ age group. Similarly, all alcohol education experiences ranging from lectures, to campaigns were given equal weights in our analysis because our survey instrument measured these experiences together. However, certain activities may have a greater impact on drinking behavior than others. Future studies should take this factor into account and attempt to give weights to these experiences or measure them separately as individual effects.

Furthermore, various sampling biases may have emerged from our surveying methods; because college students in South Korea drink large amounts of alcohol relative to adults, different patterns are likely to emerge in adult populations. Likewise, a small number of Christian colleges that were originally in our sample declined our request for participation because of their teetotalism principles and thus, had to be replaced with non-Christian colleges. Because of the face-to-face method that we employed for accuracy of obtaining responses to complicated questions, there may have been response biases, relative to social desirability. The majority of questions in our survey instrument required students to think about their drinking behaviors in the last 12 months or so, which likely resulted in recall bias.

Finally, although we included numerous lifestyle covariates as potential confounders, the limited nature and number of questions in our instrument, as well as information publicly available regarding each college campus, made it difficult for other confounding variables, relative to health, socio-demographics, gene-environment, environment, and lifestyle, to be measured and controlled for.

Despite these limitations, our study also has several strengths. Few studies have measured the effect of environmental and educational campus alcohol policies on drinking behavior for a nationally representative sample of college students in South Korea, especially with a multi-level statistical model which controls for macro-related characteristics. Our findings not only show which sub-groups are at higher risk of consuming dangerous amounts of alcohol, but show that alcohol education experience is effective only when done frequently, and in combination with environmental deterrence policies among certain subgroups (females).

## Conclusion

In conclusion, this study emphasizes the importance of prohibiting alcohol consumption in open, public college spaces, if only to prevent high-risk students from perceiving that college alcohol policies are lax. While alcohol policies and educational programs may be limited in impacting the drinking behaviors of all college students, it is undeniable that college alcohol policy is associated with student drinking behavior. It is especially important for schools to have non-judgmental and supportive mechanisms that help students with alcohol-related problems and/or AUDs (Alcohol Use Disorders) [35]. According to Blanco and colleagues, roughly 20% of college students meet the criteria for an AUD [6]. Thus, college educators and administrators should be aware that relative to students unaware of their school’s campus alcohol policy, students who believe that their college allows drinking in outdoor spaces or all areas may consume higher amounts of alcohol than their peers.

## Data Availability

Data will be made available upon request.

## Abbreviations

AUD: Alcohol Use Disorder
AUDIT-C: Alcohol Use Disorders Identification Test – Consumption
GPA: Grade Point Average
KNHANES: Korea National Health and Nutrition Examination Survey
KYRBS: Korea Youth Risk Behavior Web-based Survey
NIAAA: National Institute on Alcohol Abuse and Alcoholism
